# Enhanced spin–orbit torques by oxygen incorporation in tungsten films

**DOI:** 10.1038/ncomms10644

**Published:** 2016-02-25

**Authors:** Kai-Uwe Demasius, Timothy Phung, Weifeng Zhang, Brian P. Hughes, See-Hun Yang, Andrew Kellock, Wei Han, Aakash Pushp, Stuart S. P. Parkin

**Affiliations:** 1 IBM Almaden Research Center, San Jose, California 95120, USA; 2 Dresden University of Technology, 01062 Dresden, Germany; 3 Max Planck Institute for Microstructure Physics, 06120 Halle (Saale), Germany

## Abstract

The origin of spin–orbit torques, which are generated by the conversion of charge-to-spin currents in non-magnetic materials, is of considerable debate. One of the most interesting materials is tungsten, for which large spin–orbit torques have been found in thin films that are stabilized in the A15 (β-phase) structure. Here we report large spin Hall angles of up to approximately –0.5 by incorporating oxygen into tungsten. While the incorporation of oxygen into the tungsten films leads to significant changes in their microstructure and electrical resistivity, the large spin Hall angles measured are found to be remarkably insensitive to the oxygen-doping level (12–44%). The invariance of the spin Hall angle for higher oxygen concentrations with the bulk properties of the films suggests that the spin–orbit torques in this system may originate dominantly from the interface rather than from the interior of the films.

## Abstract (web summary)

When interfaced with a current-carrying heavy metal, spin orbit effects can generate a torque on the magnetization of a ferromagnet, understood as a bulk effect. Here, the authors show evidence of an interfacial contribution to such spin orbit torque in O-doped W/CoFeB thin film systems.

## Graphical abstract

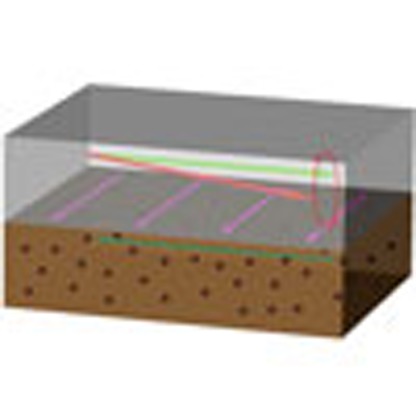

There has been considerable recent interest in the controlled manipulation of magnetic moments using spin–orbit torques for the purposes of building novel spintronic devices^1^,^2^,^3^,^4^. Spin–orbit torques are derived from pure spin currents that are generated from charge currents flowing through non-magnetic metals via spin–orbit coupling^5^,^6^,^7^,^8^. The conversion efficiency of the charge-to-spin current can be described by the spin Hall angle (SHA). Although small effects were first observed in semiconductors a decade ago (∼2 × 10^−4^ in GaAs^9^), recently much larger SHAs have been surprisingly observed in simple metals, making them potentially useful for spintronic applications. Indeed, reliable methods to measure the spin currents involve their diffusion into adjacent magnetic layers on which they can exert significant spin torques^4^,^10^,^11^. These spin–orbit torques have been shown to be sufficiently large to induce the motion of magnetic domain walls^12^,^13^,^14^,^15^,^16^, excite precessional magnetization dynamics^10^,^17^ or switch a uniform magnetic layer^3^,^4^,^18^.

Although the exact origins responsible for these spin–orbit torques in conventional metals are not understood in detail, existing theories ascribe them to combinations of intrinsic mechanisms and extrinsic spin-dependent scattering from within the bulk of the materials studied^19^. Most typically, extrinsic mechanisms will likely dominate because of innate bulk disorder in the thin films that have been studied to date. Largely neglected has been the role of interface scattering (which is known to dominate the origin of giant magnetoresistance^20^), although electronic discontinuities at interfaces between two distinct materials are known to give rise to Rashba effects that can result in charge-to-spin conversion^21^,^22^,^23^.

One of the most efficient materials exhibiting spin–orbit torques is the highly resistive β-phase of tungsten, where SHAs of up to approximately −0.35 have been reported^24^,^25^,^26^. In contrast, the α-phase of tungsten exhibits a much smaller SHA. Consequently, one would expect that the SHA should scale strongly with significant changes in the microstructure of the materials under consideration. A detailed understanding of the role of materials' microstructure on the spin–orbit torques is thus crucial for enhancing the efficiency of these effects, as well as giving insight towards the underlying mechanisms responsible for the spin–orbit torques.

Here we demonstrate that by doping oxygen into tungsten thin films, large spin–orbit torques are attained that are rather insensitive to considerable changes in the resistivity and film microstructure. The spin–orbit torques that we observe, when quantified as a SHA, are the largest to be reported thus far for a conventional metal-based system (approximately −0.5), and could be of considerable technological interest for spintronic applications. Furthermore, our findings suggest that these very-efficient spin–orbit torques are largely interfacial in origin.

## Results

### Spin torque ferromagnetic resonance of W(O) | CoFeB structures

**Figure 1:**
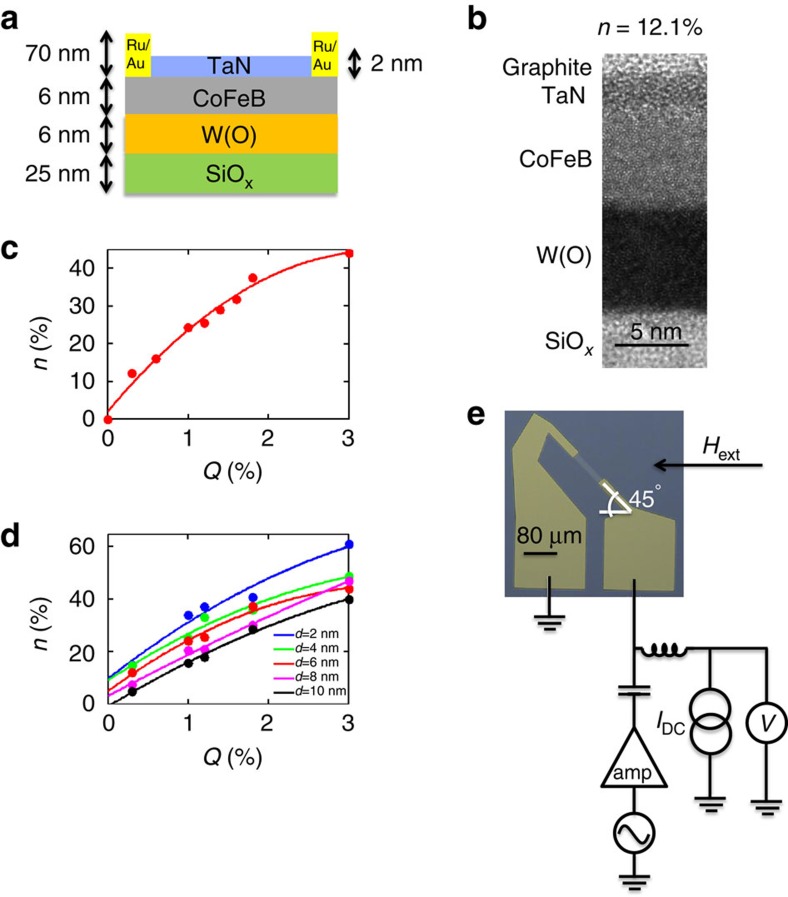
STFMR measurement. (**a**) Device structure consisting of substrate | W(O) (6) | Co_40_Fe_40_B_20_ (6) | TaN (2) (with film thickness in parantheses in nm). (**b**) Cross-section transmission electron microscope (TEM) image of the device structure for *n*=12.1% (*Q*=0.3%). (**c**) Oxygen concentration *n* is plotted against gas flow *Q* as determined by Rutherford backscattering spectrometry (RBS). (**d**) Oxygen concentration versus gas flow *Q* for W(O) films of different thicknesses. (**e**) Microscope image and schematic of the electrical circuit used for STFMR measurements.

The films in this study were deposited at room temperature using d.c. magnetron sputtering onto oxidized silicon substrates consisting of the layer-structure Si substrate | SiO_*x*_ (25) | W(O) (6) | Co_40_Fe_40_B_20_ (6) | TaN (2) (Fig. 1a,b; with film thicknesses in nm in parentheses). Hereafter, we shall refer to oxygen-doped tungsten as W(O) and Co_40_Fe_40_B_20_ as CoFeB. The amount of oxygen gas flow in the reactive mixture during the sputtering, *Q*, was varied between 0 and 3%. A 20-Å-thick highly resistive TaN layer (5.08 Ωcm for 50 nm thickness) was used for capping the magnetic CoFeB film (109 μΩ cm for 6 nm thickness; Fig. 1a,b). The TaN layer was ∼10^4^ times more resistive than the most resistive W(O) film considered in this study, ensuring that current primarily flows in the W(O) and CoFeB layers. The atomic oxygen concentration *n* was determined in films specially deposited on graphite substrates using Rutherford backscattering spectrometry (see Fig. 1c). *n* increases monotonically with increasing gas flow *Q* reaching ∼44 atomic % at the maximum oxygen gas flow used, for 6-nm-thick W(O) films. Furthermore, we found that the amount of oxygen incorporation was increased for thinner films (Fig. 1d). This finding is consistent with previous studies of sputtered tungsten thin films, and one explanation for it may arise from the compressive stress, which increases with film thickness for W films deposited at comparable pressures to ours^27^. The increased compressive stress leads to a smaller lattice constant and hence reduced oxygen incorporation.

We use the spin transfer torque (STT) ferromagnetic resonance technique^10^ to determine an effective SHA, 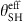, which we use to assess the value of the damping-like spin–orbit torque. Figure 1e shows a microscope image of a representative microstrip device (10 μm × 80 μm) with a 45° tilt (for more information on the fabrication, see Methods) and a schematic illustration of the experimental set-up used for this measurement. A radiofrequency (RF) current is passed through the device, which generates spin–orbit torques as well as an Oersted field, in the presence of an externally applied magnetic field *H*_ext_. These torques cause a sustained precession of the magnetization of the magnetic layer, which is measured through the mixing d.c. voltage *V*_mix_ that is generated from the oscillating anisotropic magnetoresistance and spin Hall magnetoresistance^28^ signals and the applied RF current. The RF source has been set to an output power of 5 dBm and then amplified to 22 dBm. We extract the magnitude of 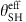 by performing two different types of analyses based on fitting *V*_mix_ to a Lorentzian function consisting of symmetric (*F*_S_) and asymmetric (*F*_A_) components:


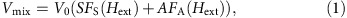


where


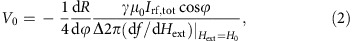



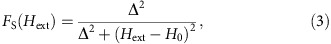


and


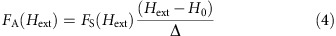


**Figure 2:**
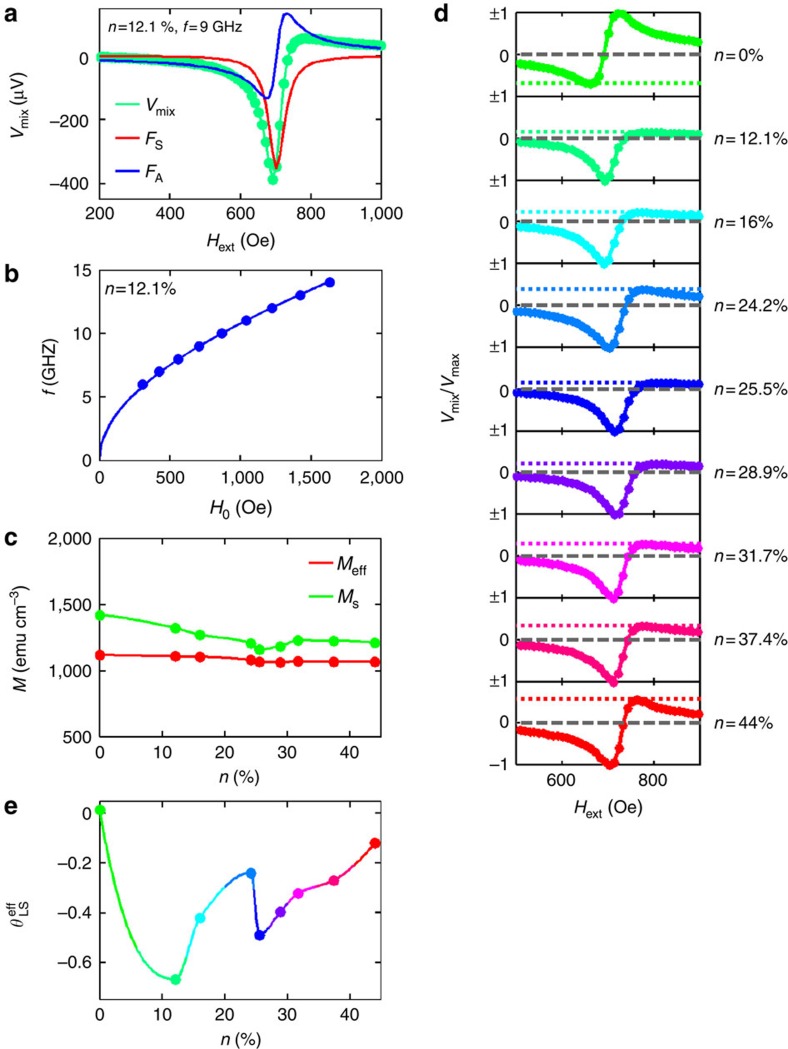
Line-shape analysis. (**a**) *V*_mix_ along with the fitted (green), symmetric (*F*_S_, red) and asymmetric (*F*_A_, blue) Lorentzian functions used for the fitting for *n*=12.1% (*Q*=0.3%). (**b**) Frequency as a function of the resonant field for *n*=12.1% (*Q*=0.3%) used in the Kittel formula fitting. (**c**) The magnetization *M*_S_ determined by VSM and effective demagnetization field *M*_eff_ from the Kittel formula fitting versus oxygen concentration *n*. (**d**) *V*_mix_ normalized to either its minimum or maximum value for different oxygen concentrations at 9 GHz. The coloured dotted line denotes the corresponding maximum/minimum value of *V*_mix_. The dotted line (dashed grey line) indicates the zero level. (**e**) SHA calculated from the line-shape analysis 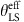 as a function of the oxygen concentration *n*.

Here Δ is the linewidth and *H*_0_ is the resonance field. The prefactors, *S* and *A*, are the contributions of the symmetric and asymmetric parts, respectively, and *V*_0_ is a constant prefactor. In equation (2), *R* represents the resistance of the device, which, because of a combination of anisotropic magnetoresistance and spin Hall magnetoresistance, depends on the angle *ϕ* between the current and the magnetization (which for our measurements is either at 45° or −135°, depending on the applied field direction), *γ* is the gyromagnetic ratio, *μ*_0_ is the magnetic permeability of free space, *I*_rf,tot_ is the RF current through the device and *f* is the frequency of the applied RF current. Figure 2a shows representative data for *V*_mix_ (*n*=12.1%, *Q*=0.3%) for an RF current applied at 9 GHz, along with the symmetric and asymmetric components of the data extracted from the fitting.

The first type of analysis, which we call here the line-shape analysis 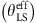, associates the symmetric part of the Lorentzian function to the antidamping-like STT and the asymmetric part to the Oersted field in the system. Meanwhile, the line-width analysis is based on measuring changes in the linewidth 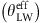, that is, damping, as a function of a superimposed d.c. current. We note that the SHAs we report here are effective values because we do not take into account the transparency^29^, whose role is minor in the materials under consideration here because of their relatively high resistivities.

### Line-shape analysis

The determination of the SHA based on the line-shape analysis is described by the following formula^10^:


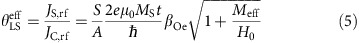



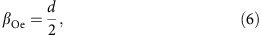


where *e* represents the electron charge and *ħ* represents Planck's constant above. We deduce the thicknesses of the magnetic (*t*) and non-magnetic (*d*) layers by determining the film deposition rates from measurements of the thicknesses of nominally 50-nm-thick calibration films with a profilometer. The saturation magnetization *M*_S_ was measured by vibrating sample magnetometry (VSM) and the effective demagnetization field *M*_eff_ was calculated by fitting the frequency versus the resonance field, according to the Kittel formula (Fig. 2b; see Supplementary Table 1 for a summary of the parameters above for the films under study):


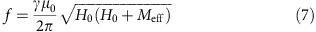


Figure 2c illustrates a weak decrease in *M*_S_ with increasing oxygen concentration.

Figure 2d shows the fitting of normalized *V*_mix_ (*H*_ext_) at 9 GHz for different amounts of oxygen incorporated into the W(O) film. The data are normalized either to the minimum or maximum value of *V*_mix_. The corresponding maximum/minimum of *V*_mix_ is indicated by the coloured dotted line, as a guide to the eye. Using the data analysis given above, large SHAs imply a large symmetric component relative to the asymmetric component. Consequently, the closer the dotted line is to zero (grey dashed line in figure), the higher the SHA is. The highest value we reach with this analysis is 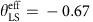. Moreover, the variation of the SHA as a function of the oxygen concentration is rather significant (Fig. 2e).

We further note that this commonly used analysis technique requires careful attention from possible artefact voltages. In particular, the line-shape analysis assumes that the symmetric component arises completely from the antidamping STT. However, a contribution to the symmetric component from spin pumping^30^ that induces an inverse spin Hall effect^31^ voltage can occur that can diminish and even change the sign of the observed SHA, which is what we find for *n*=0% (see Supplementary Note 1 and Supplementary Figs 1 and 2). Another caveat of this analysis is that the asymmetric component is assumed to arise completely from the Oersted field-generated torque; however, for the *n*=0% sample, we find this torque to be smaller than the field-like STTs (see Supplementary Note 1 and Supplementary Fig. 2). Thus, a complete quantitative analysis based on this technique requires accounting for the field-like STTs as well as a consideration of the size of the spin-pumping contribution to the symmetric signal. Nevertheless, we perform the line-shape analysis to gain a qualitative sense of the dependence of the SHA with the oxygen concentration in our films, but use a line-width analysis to more reliably assess the value of the SHA that is generated solely from the antidamping STT.

### Line-width analysis

**Figure 3:**
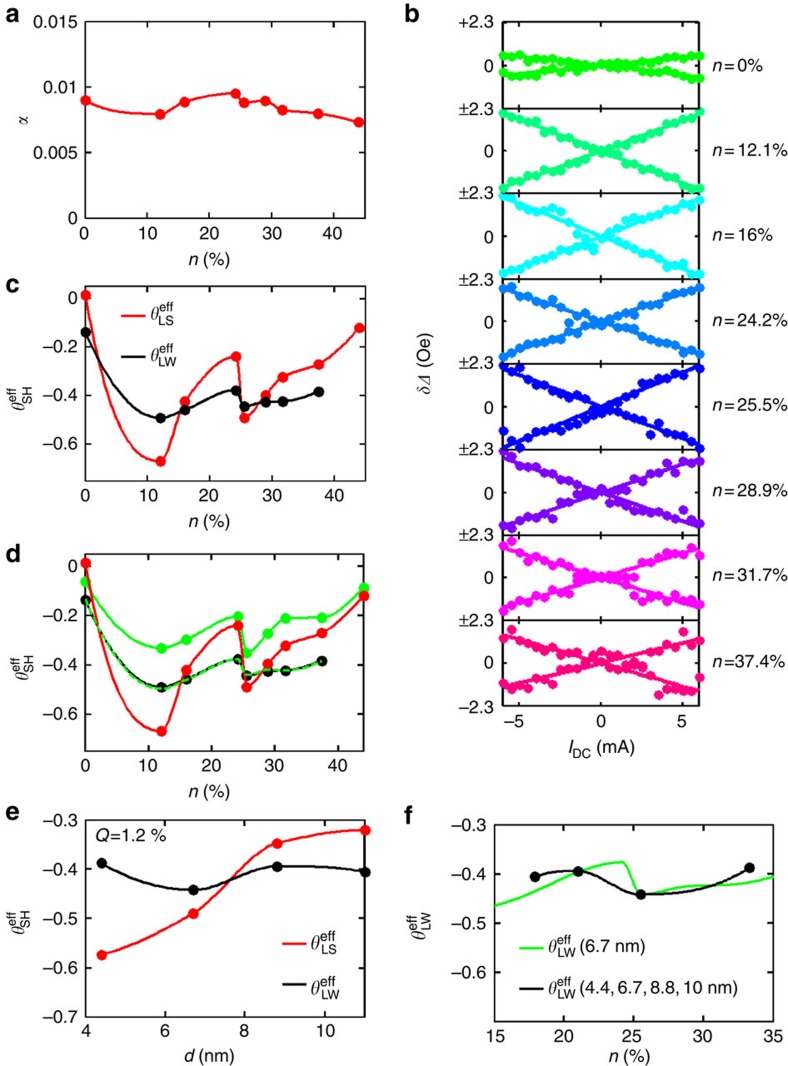
Line-width analysis. (**a**) Dependence of the Gilbert damping on *n*. (**b**) Change of linewidth (*δ*Δ) versus d.c. current *I*_d.c._ for different *n*. (**c**) Damping-like spin Hall angle 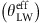 versus *n* (black curve) calculated from d.c.-current-dependent linewidth. 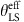 (red curve) is shown for comparison purposes. (**d**) Correction of 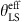 (red) with the RF current measured with a network analyser (green curve, 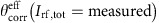) and correction with a slightly adjusted RF current (dashed green curve, 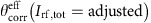) to match the black curve 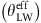. (**e**) Thickness dependence of 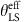 and 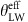 for *Q*=1.2%. (**f**) 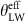 for ∼6.7-nm-thick films as a function of oxygen concentration *n*, and 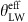 of films with *Q*=1.2% of various thicknesses.

The Gilbert damping *α* can be deduced from the dependence of Δ on frequency that is given by


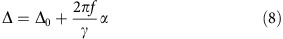


where Δ_0_ represents the inhomogeneous broading. We observe that the Gilbert damping remains relatively constant with respect to the oxygen concentration, with an average value of *α*∼0.0085 (Fig. 3). We have also compared the change in the Gilbert damping with the presence of the W(O) layer to deduce the effective spin-mixing conductance as a function of *n*, and find it to be relatively constant (see Supplementary Note 2 and Supplementary Fig. 3).

We further measured the dependence of Δ on an applied d.c. current through the device (Fig. 3b)


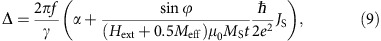


To determine 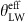 originating from the antidamping-like STT^10^,^32^ one has to use the slopes *δ*Δ/*δI*_d.c._ from Fig. 3b:


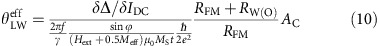


*R*_FM_ and *R*_W(O)_ are the resistances of the ferromagnetic layer and W(O) layer, respectively, and *A*_C_ is the cross-sectional area of the device. From this line-width analysis we find that 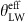 increases abruptly on the introduction of oxygen, but varies slightly with the addition of more oxygen into the tungsten film, as shown in Fig. 3c. Although the dependence of 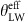 and 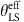 on oxygen concentration is qualitatively similar, the variation in 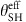 with higher oxygen concentrations is much more pronounced when using the line-shape analysis (Fig. 2e). The SHA values reached here are at most 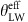
=−0.49 where *n*=12.1%. The SHA in the pure tungsten film, by comparison, is 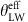
=−0.14, which is consistent with a previous report for a 6-nm-thick film where both the α- and β-phase tungstens were found to co-exist^24^. The difference between the line-shape and line-width analyses stems mostly from the contributions of the spin-pumping and field-like torques. To quantitatively reconcile the values based on the two analysis techniques, an accurate determination of the RF current through the device is required (see Supplementary Note 1 and Supplementary Figs 1 and 2)^33^,^34^,^35^. Figure 3d shows the comparison between the line-shape and line-width analyses after accounting for the spin-pumping and field-like torques. Here we shall focus on the line-width analysis when drawing conclusions regarding the SHA.

The role of thickness of the W(O) on the SHA was examined for a gas flow *Q*=1.2% (Fig. 3e). However, one important point to note is that the amount of oxygen that is actually incorporated in the film varies significantly with the thickness of the grown film, as mentioned above (Fig. 1d). Thus, the SHA of these films can be plotted as a function of oxygen content, irrespective of their thickness, and compared with data from above where the thickness remains constant and the oxygen content is intentionally varied (Fig. 3f). On the basis of this analysis, we find that the SHA does not change significantly despite large changes in thickness. Furthermore, based on a volume origin of the SHE, the SHA as a function of thickness varies as


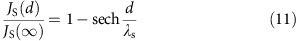


Our data thus imply that either the spin diffusion length *λ*_S_ would have to be significantly smaller compared with 4.4 nm or alternatively the spin–orbit torque has an interfacial origin.

### Material characterization

**Figure 4:**
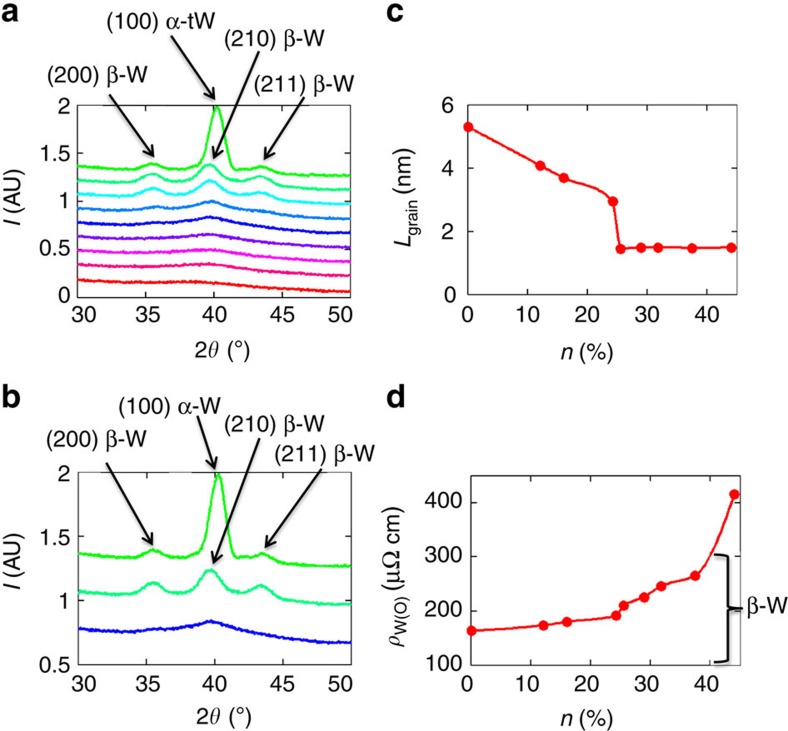
Materials' characterization. The colour schemes used here for *n*, indicating the oxygen concentration, are the same as in Figs 2 and 3. (**a**) X-ray diffraction for W(O) films with different *n*. The crystallographic orientation and the corresponding phase (α or β) are indicated by arrows. (**b**) Detailed X-ray diffraction on films with three noteworthy oxygen concentrations: pure W, *n*=12.1% and *n*=25.5%. There is a small shift in the peak at 2*θ*=40° from *n*=0% to *n*=12.1%, indicating a larger amount of β−W for *n*=12.1%. (**c**) Grain size *L*_Grain_ versus *n*. (**d**) Resistivity versus *n*. β−W has a resistivity between 100 and 300 μΩ cm as indicated by the bracket.

To understand the origin of the observed trends in the SHA, we performed resistivity and X-ray diffraction measurements. To increase the signal-to-noise ratio, X-ray diffraction was performed on multilayers with the stack sequence 4·[SiO_*x*_ (2.5) | W(O) (6) | CoFeB (6) | TaN (2.5)] (with film thicknesses in parenthesis in nm). The X-ray diffraction measurements (Fig. 4a,b) reveal that the tungsten film grown without any oxygen exhibits a predominantly α−W phase with a (100) orientation (strong peak at 2*θ*=40.3°) with some contributions from a β−W structure with (200) and (211) crystal orientations (2*θ*=35.4° and 43.7°, respectively). The sample with the highest SHA, corresponding to *n*=12.1%, exhibits a peak at 2*θ*=39.8° that is distinct from the α−W (100) peak and corresponds to the (210) peak for β−W. Thus, the X-ray data show that the amount of β−W is increased significantly for *n*=12.1% compared with *n*=0% and there is no evidence for any remaining α−W. The X-ray diffraction measurements also show that increasing the oxygen concentration in the films leads to eventually significantly broader and weaker X-ray diffraction peaks, indicating an increasing nanocrystallization of the W(O) films. The grain size can be calculated using the Scherrer equation


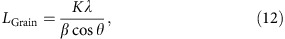


with *K*=1 (shape factor), wavelength *λ*=0.154 nm, *β* being the half intensity width and *θ* being diffraction angle. The grain size versus oxygen concentration is shown in Fig. 4c. Interestingly, we observe a sudden decrease in the grain size at *n*=25.5% to an oxygen concentration-independent grain size for higher *n*. The transition indicates that the W(O) material becomes nanocrystalline at a critical *n*. The X-ray diffraction measurements of the actual films used in the STT ferromagnetic resonance technique study as well as much thicker films, nominally ∼50 nm thick, are shown in the Supplementary Section, and show similar trends to the multilayer films (see Supplementary Note 3 and Supplementary Fig. 4).

We further investigated the dependence of the resistivity on the oxygen concentration in our W(O) films (Fig. 4d). The resistivity of Si substrate | SiO_*x*_ (25) | W(O) (6) | TaN (2) (with film thicknesses in parenthesis in nm) films were measured using a four-point measurement. The resistivity shows a continuous increase with an increasing oxygen content. However, we note that the resistivity does not increase significantly until the films become nanocrystalline. Moreover, there is a sharp increase in resistivity (at *n*∼37%). We speculate that this could arise from the formation of an oxygen network, which encloses metallic tungsten grains (causing the resistance to suddenly increase). The resistivity of β−W is in the range of 100–300 μΩ cm (ref. 24), whereas α−W is 11.2 μΩ cm, which corroborates our X-ray diffraction measurements that indicate that the 6-nm-thick pure tungsten film contains β−W. We have also performed a resistance versus temperature study to investigate transport mechanisms underlying the observed effects (see Supplementary Note 4 and Supplementary Fig. 5)^26^,^36^. With an increasing oxygen content, 50-nm-thick films become less metallic, until for when *n* is greater than ∼25.5%, the film resistance increases with a decreasing temperature. For the thinner films, the resistance increases with decreasing temperature for all films. For *n*<31.7%, indications of superconductivity are observed below ∼2 K that give strong evidence of the A15 phase that is known to exhibit a superconducting transition near this temperature.

## Discussion

Our materials' characterization shows that the incorporation of oxygen stabilizes the β−W. This is consistent with previous studies, which have shown that the formation of β−W is extremely sensitive to the oxygen incorporation during the growth process^37^,^38^,^39^,^40^,^41^, and an oxygen concentration of only 10% is sufficient to stabilize β−W. In our experiments, at *n*=12.1%, we obtain a SHA of 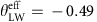, which is higher than any previous measurements in conventional metal-based systems.

With the addition of further oxygen-doping, we observe the formation of a nanocrystalline structure. Despite the large changes we have observed in the bulk microstructure (as evidenced by a grain size change by more than a factor of 2), and a resistivity change by a factor of 2, we see that the SHA still remains very large. Our observation of the independence of the SHA despite significant changes in the bulk properties, namely the oxygen concentration, the resistivity, the microstructure as well as thickness, suggests that the mechanism responsible for the observed highly efficient spin–orbit torque may originate at the W(O)/ferromagnet interface. Our observations are consistent with theoretical proposals of antidamping-like torques from interfacial spin–orbit coupling such as the Rashba effect^42^,^43^,^44^,^45^,^46^. Such interfacial spin–orbit torques have also been reported in the Pt | oxidized CoFeB system^47^. One important difference in our experiment is that we do not intentionally oxidize the CoFeB. Furthermore, W is a heavy metal with large spin–orbit coupling, which gives rise to a large Rashba effect in combination with a 3*d* ferromagnet, such as CoFeB (ref. 48). For the Gd(0001) surface, it has been shown that on oxidation the Rashba parameter was enhanced^49^. Such a phenomenon may also play a role in our experiments. It has been postulated that spin memory loss (SML) at the interface could reduce the magnitude of the spin current^50^. It is difficult to estimate the magnitude of any SML but from the weak dependence of *G*_eff_ on oxygen content (Supplementary Fig. 3), it therefore follows that the SML would also be weakly dependent on the oxygen content, and, therefore cannot account for the variation of effective SHA with oxygen content that we observe. In any case, any contribution from SML would simply mean that the values of the SHA that we report would be even larger. We have also examined the role of the inhomogeneous broadening on the SHA, and observe that it is uncorrelated to any changes we observe in the SHA (see Supplementary Note 5 and Supplementary Fig. 6).

We thus postulate that the first SHA peak at *n*=12.1% is caused by β-phase stabilization and the large SHA observed at the higher oxygen concentrations to arise from interfacial spin–orbit torques. It is still possible, however, that for *n*=12.1% there is already a considerably large interfacial spin–orbit torque that explains why we obtain larger values for β−W compared with previous studies. Furthermore, we note that one alternative explanation for the large SHA at high oxygen content may arise from amorphization of the W(O) material (Fig. 4c) and the SHA could be enhanced because of extrinsic effects in analogy with the large anomalous Hall effects found in amorphous magnetic materials^51^. However, in this case, one would expect the SHA to increase with higher oxygen concentration and scale with the resistivity. We instead observe a nearly constant SHA towards very high oxygen concentrations and thus believe that it is more likely that interfacial spin–orbit torques account for our observations.

Our results illustrate an intriguing path towards enhancing the magnitude of spin–orbit torques, and also serve to bridge the link between oxide electronics with spintronics.

## Methods

### Sample growth and preparation

The films were sputtered in the presence of Argon gas on undoped oxidized silicon with (100) orientation. Two-inch-diameter targets were used with the target to substrate distance kept fixed at 12.5 cm. The base pressure before deposition was less than 10^−8^ torr and the pressure during deposition was 3 mTorr. The W(O) layers were formed by introducing oxygen gas into the Ar-sputtering gas atmosphere during the film deposition. The gas flow of oxygen *Q* was varied between 0 and 3%. The highly resistive TaN-capping layer was fabricated by introducing 50% Nitrogen into the Argon gas flow. Device-patterning was performed with optical lithography and ion milling, followed by a 14-nm-thick AlO_*x*_ refill. Subsequently, 5 nm ruthenium and 65 nm gold were deposited and patterned for electrical contacts to the device.

## Additional information

**How to cite this article:** Demasius, K.-U. *et al*. Enhanced spin–orbit torques by oxygen incorporation in tungsten films. *Nat. Commun.* 7:10644 doi: 10.1038/ncomms10644 (2016).

## Supplementary Material

Supplementary Information Supplementary Figures 1-6, Supplementary Tables 1-2, Supplementary Notes 1-5 and Supplementary References
