# Supplementary material for: Enhanced spin–orbit torques by oxygen incorporation in tungsten films
Source: Nat Commun. 2016 Feb 25;7:10644. doi: 10.1038/ncomms10644 (PMC4773389; doi:10.1038/ncomms10644)
Supplement: Supplementary Information — Supplementary Figures 1-6, Supplementary Tables 1-2, Supplementary Notes 1-5 and Supplementary References [file ncomms10644-s1.pdf]

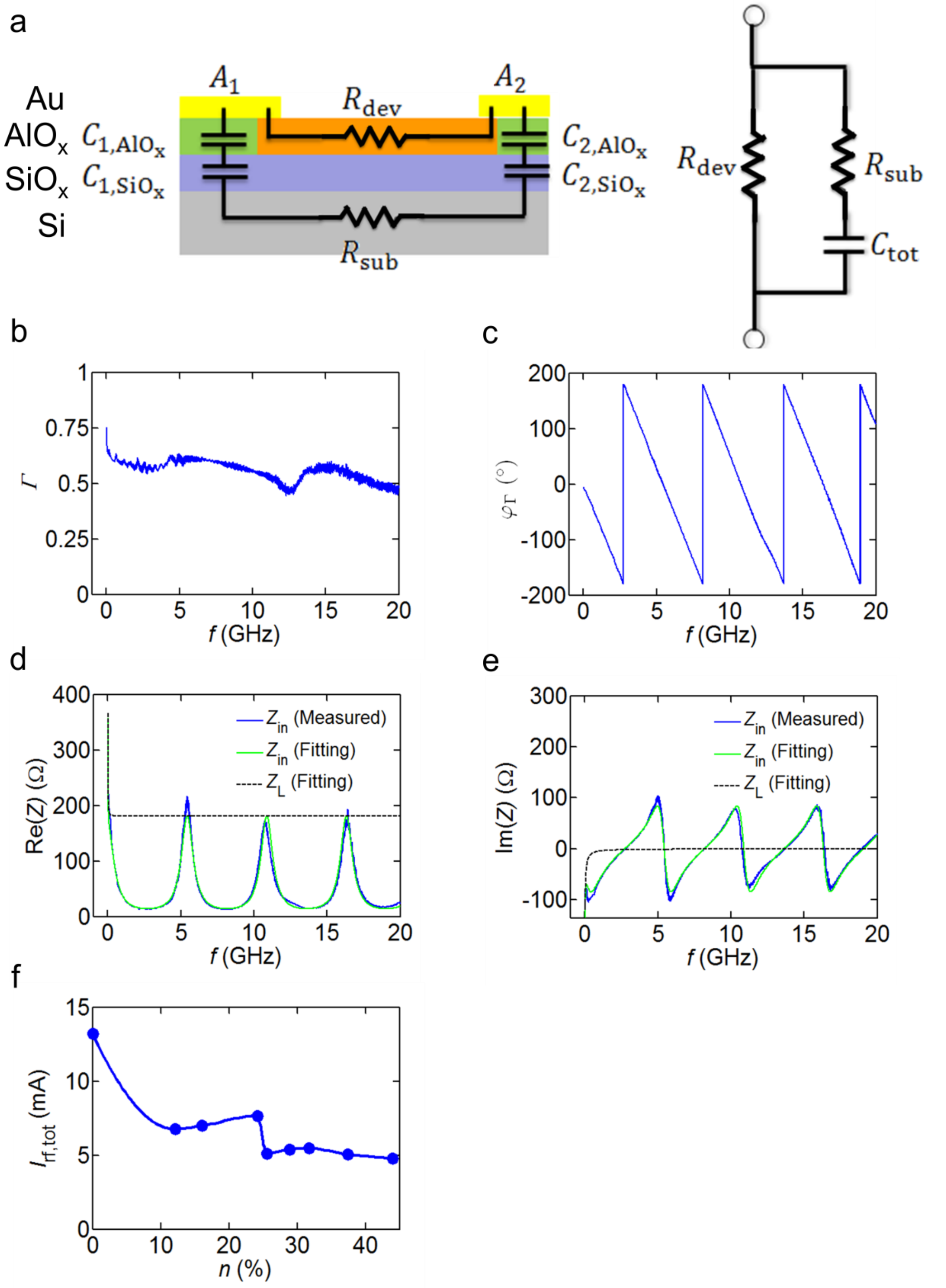

Supplementary Fig. 1: **RF current determination for 9 GHz.** a) Structure of device and gold pads with parasitic substrate coupling and circuit model. b) Reflectance for $n = 0$ % and c) phase for $n = 0$ % measured with network analyzer. d) Measured real part of impedance from

reflectance (blue), fitting to the circuit model (green) and impedance $\underline{Z_{\mathrm{L}}}$ neglecting the transmission line modulation. e) Measured imaginary part of impedance from reflectance (blue), fitting to the circuit model (green) and impedance $\underline{Z_{\mathrm{L}}}$ neglecting the transmission line modulation. f) RF current through the device resistance R_{dev} versus oxygen content.

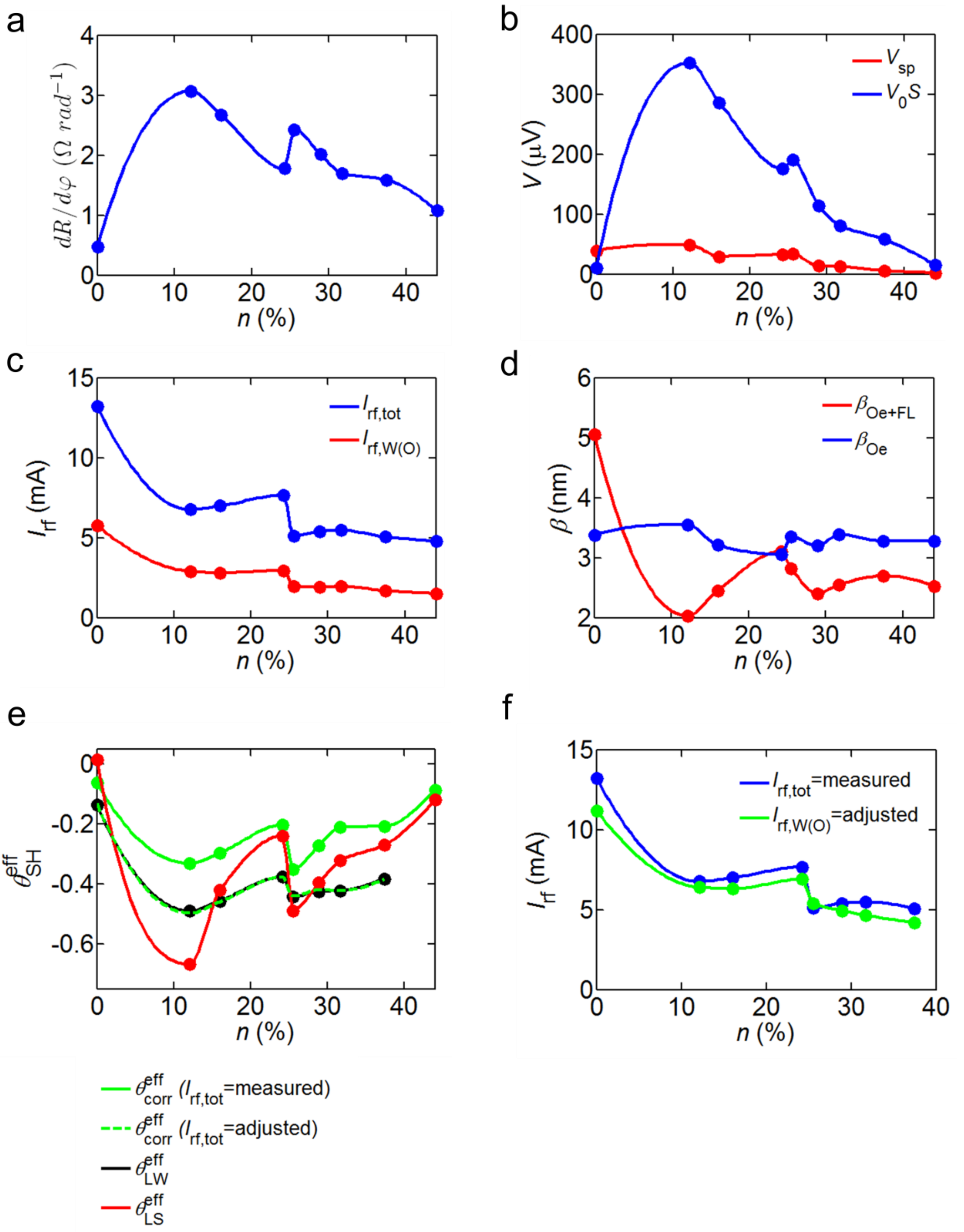

Supplementary Fig. 2: **Spin pumping and field like torque contributions of STFMR signal.** a) $dR/d\varphi$ determined by AMR/SHMR measurement versus oxygen content. b) Spin pumping voltage in comparison to symmetric voltage of V_{mix}. c) RF current through tungsten layer $I_{\mathrm{rf,W(O)}}$ in comparison to total RF current through device $I_{\mathrm{rf,tot}}$. d) β factor from the

Oersted field, β_{Oe}, in comparison to β factor from Oersted and field like torque, $\beta_{\mathrm{Oe+FL}}$. e) SHA from $\theta_{\mathrm{LS}}^{\mathrm{eff}}$ (red), $\theta_{\mathrm{LW}}^{\mathrm{eff}}$ (black), and $\theta_{\mathrm{LS,corr}}^{\mathrm{eff}}$ (green). The dashed green curve shows $\theta_{\mathrm{LS,corr}}^{\mathrm{eff}}$, uses the current values shown in f). f) A comparison between the $I_{\mathrm{rf,tot}}$ from Supplementary Fig. 1f with the RF currents that are necessary to equate $\theta_{\mathrm{LS}}^{\mathrm{eff}}$ and $\theta_{\mathrm{LW}}^{\mathrm{eff}}$.

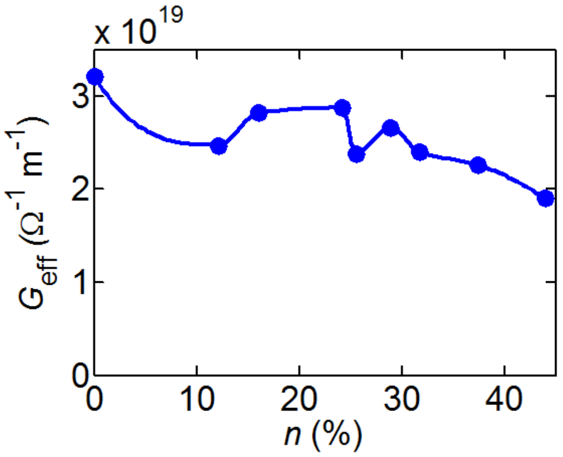

Supplementary Fig. 3: **Effective spin mixing conductance.** Effective spin mixing conductance G_{eff} versus oxygen content.

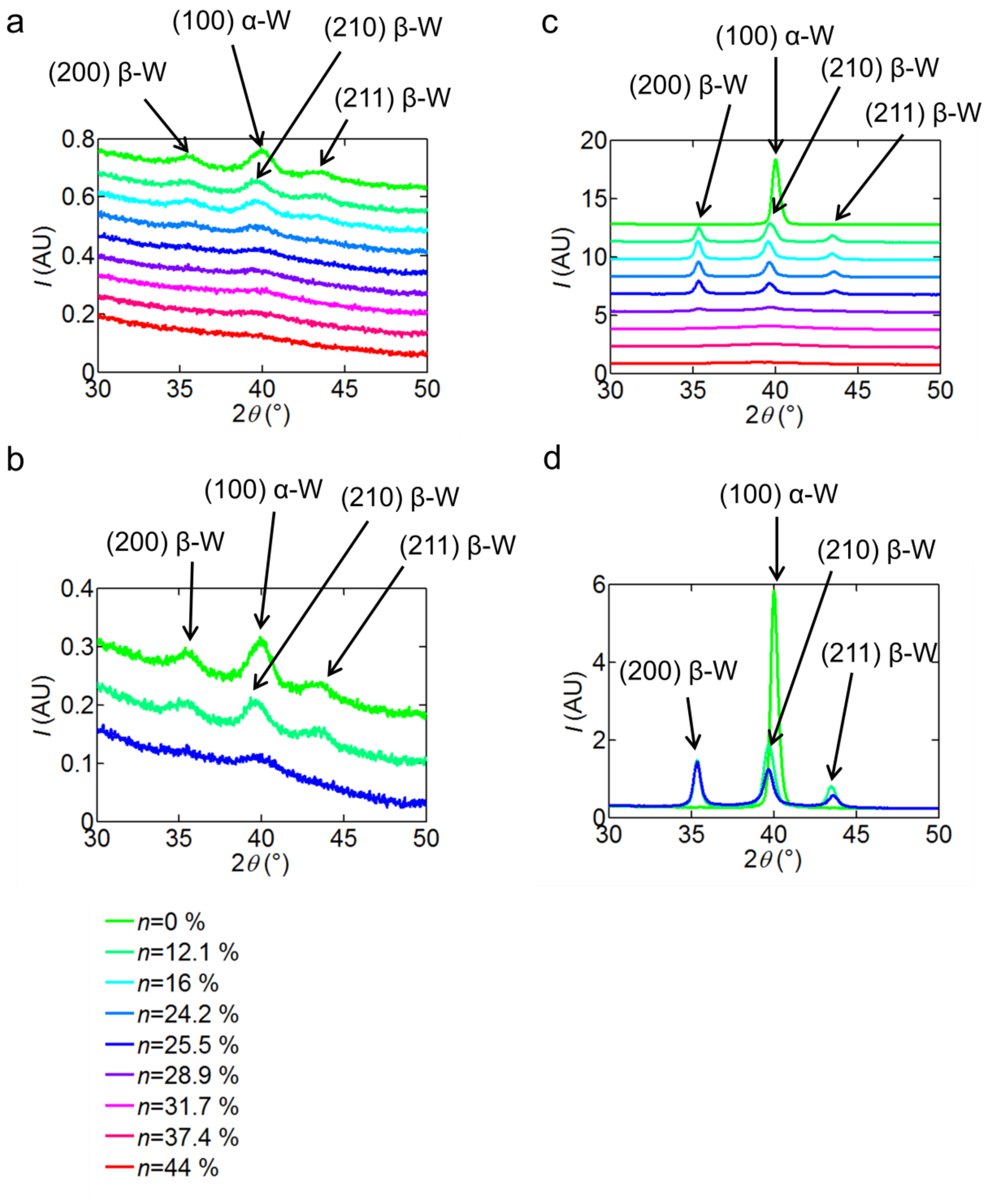

Supplementary Fig. 4: **XRD data for thick and thin films.** a) XRD of thin (6 nm) ST-FMR films for all oxygen contents. b) Subset of XRD of thin (6 nm) ST-FMR films for three noteworthy oxygen contents. c) XRD of thick (50 nm) calibration films for all oxygen contents. d) Subset of XRD of thick (50 nm) calibration films for three noteworthy oxygen contents.

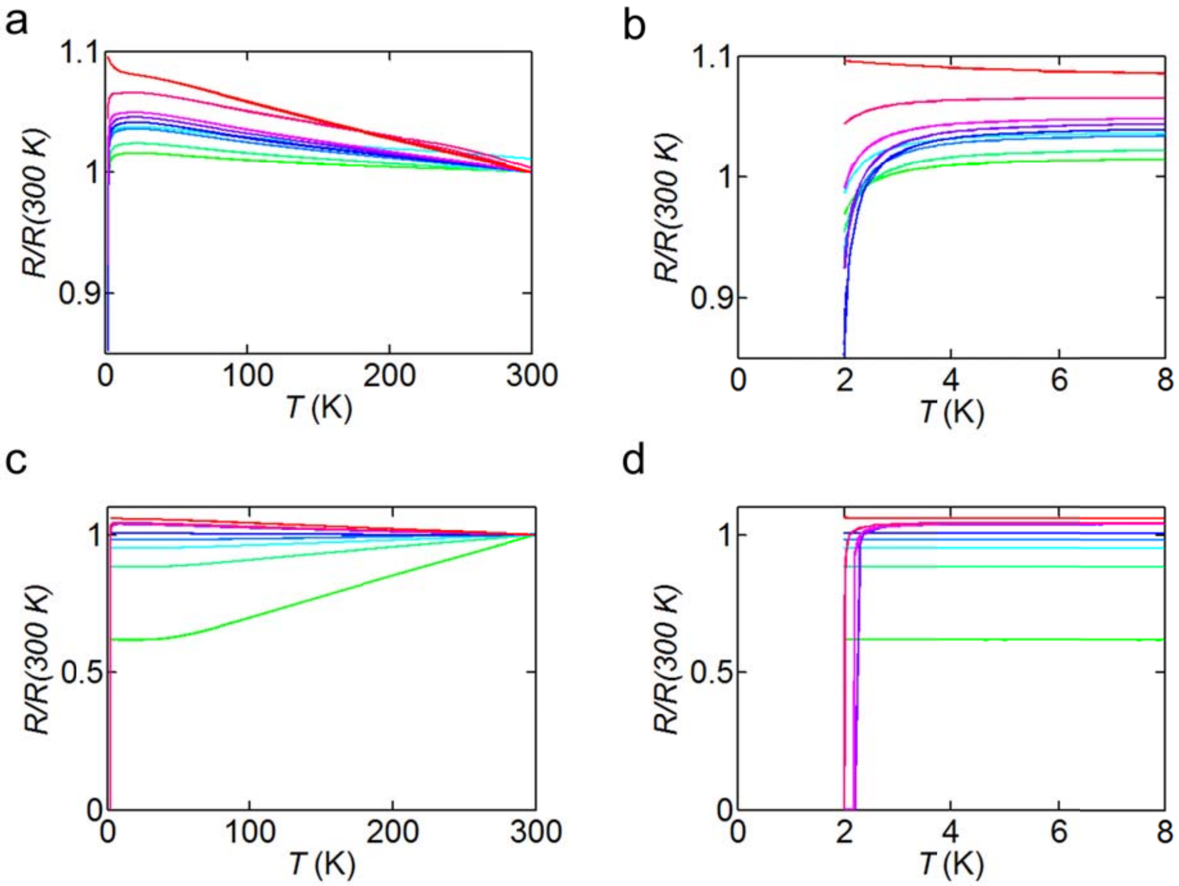

Supplementary Fig. 5: **Resistance versus Temperature.** The colors used here denote the same oxygen concentrations as in Supplementary Fig. 4. a) Normalized resistance versus temperature (0-300 K) for thin (6 nm) W(O) films. b) Zoom in of normalized resistance versus temperature (0-8 K) for thin (6 nm) W(O) films. c) Normalized resistance versus temperature (0-300 K) for thick (50 nm) W(O) films. d) Zoom in of normalized resistance versus temperature (0-8 K) for thick (50 nm) W(O) films.

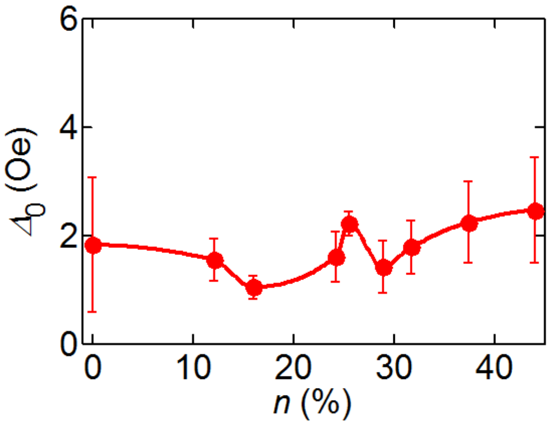

Supplementary Fig. 6: **Inhomogeneous broadening.** Inhomogeneous broadening versus oxygen content.

Q (%)	n (%)	(200) β-W L_{grain} (nm) (35.42 °)	(211) β-W L_{grain} (nm) (43.65 °)	(210) β-W L_{grain} (nm) (39.75 °)	(100) α-W L_{grain} (nm) (40.31 °)	Average L_{grain} (nm)
0	0	5.86	4.63	-	7.23	5.90
0.3	12.1	4.83	4.50	4.30	-	4.54
0.6	16	4.41	4.00	3.90	-	4.11
1.0	24.2	3.52	3.00	3.33	-	3.28
1.2	25.5	-	-	1.62	-	1.62
1.4	28.9	-	-	1.65	-	1.65
1.6	31.7	-	-	1.66	-	1.66
1.8	37.4	-	-	1.64	-	1.64
3.0	44.0	-	-	1.65	-	1.65

Supplementary Table 1: **XRD Data.** The oxygen gas flow, Q, used during the sputter deposition of the W(O) film and the atomic oxygen concentration (n) of the same film determined by RBS is tabulated, along with the diffraction angle (2θ) and the corresponding

grain size L_{grain} for each of the salient peaks. The grain size was calculated using the Scherrer equation, and the average grain size is also listed.

Q (%)	n (%)	M_S (emu cm^{-3})	M_{eff} (emu cm^{-3})	$\theta_{\text{LS}}^{\text{eff}}$	$\theta_{\text{LW}}^{\text{eff}}$	$\rho_{W(O)}$ ($\mu\Omega\ cm$)	d (nm)
0	0	1420.36	1118.97	0.015	-0.14	164.23	6.75
0.3	12.1	1322.11	1106.59	-0.67	-0.49	173.79	7.1
0.6	16	1270.89	1104.02	-0.42	-0.46	180.16	6.42
1.0	24.2	1207.36	1079.64	-0.24	-0.38	192.09	6.1
1.2	25.5	1158.21	1068.27	-0.49	-0.44	210.27	6.7
1.4	28.9	1184.56	1061.49	-0.40	-0.43	226.31	6.40
1.6	31.7	1226.99	1069.49	-0.32	-0.42	246.04	6.77
1.8	37.4	1226.16	1068.04	-0.27	-0.38	265.90	6.56
3.0	44.0	1210.10	1068.45	-0.12		416.24	6.56

Supplementary Table 2: **Magnetic and electrical Properties.** Summarizes the spin Hall angle determined based on both the line-shape ($\theta_{\text{LS}}^{\text{eff}}$) and line-width ($\theta_{\text{LW}}^{\text{eff}}$) analysis, resistivity of the W(O) film $\rho_{\text{W(O)}}$, saturation Magnetization M_S and effective magnetization M_{eff} of the CoFeB film, along with the thickness (d) of the W(O) film.

Supplementary Note 1: Influence of spin pumping and field like torque on Lineshape analysis

In order to explain the difference between the line shape and line width analysis, we estimated the contributions of the spin pumping voltage and field like torque to the signals we obtain from the line shape analysis ($\theta_{\mathrm{LS}}^{\mathrm{eff}}$) for 9 GHz.[1–3] Estimating these parameters requires knowing the actual RF current running through the device, which we deduce from the reflectance (S_{11}) parameter that is measured with a network analyzer. We also note that parasitic circuit elements which shunt a portion of the RF current through our device must also be accounted for. The most dominant source of this shunting lies in capacitive coupling through the substrate.

Supplementary Fig. 1a shows the cross-sectional view of the device, and shows sources of parasitic impedances. Since the gold pads have different areas, they act as different capacitances (C_1 and C_2) and these capacitances are connected in series via a substrate resistance R_{sub}. The capacitances C_1 and C_2 are composed of the AlO_x capacitances from the refill process and SiO_x capacitances from the substrate. We can model the entire circuit with the device resistance in parallel with an effective total shunting capacitance, C_{tot} in series with the substrate resistance R_{sub}. From the dimensions of the microscope image in Fig. 1e of the main paper, one can estimate the value of $C_{\mathrm{tot}} \approx 2 \cdot 10^{-11}$ F and that of the substrate resistance $R_{\mathrm{sub}} \approx 533\ \Omega$ ($\rho_{\mathrm{sub}} = 20\ \Omega \cdot cm$). The device resistance R_{dev} is ascertained based on DC resistance measurements. R_{sub} and C_{tot} are also empirically determined based on the network analyzer measurements. Supplementary Figs. 1b and 1c show representative data for the reflectance and phase measured by the network analyzer for the sample with $n = 0$ %. The phase shows a periodic behaviour, which is caused by RF standing waves from the high frequency probes with length l (We calibrate our network analyser up to our probes). From

the reflectance $\underline{\Gamma}$, it is possible to calculate the impedance $\underline{Z_{\mathrm{in}}}$ (underlined symbols indicate complex numbers):

$$\underline{Z_{\mathrm{in}}} = Z_0 \frac{1 - \underline{\Gamma}}{1 + \underline{\Gamma}} \qquad (1)$$

With $Z_0 = 50\ \Omega$ being the reference impedance. The real and imaginary part of $\underline{Z_{\mathrm{in}}}$ are shown in Supplementary Fig. 1d and e (blue curve). The peaks here correspond to standing waves on the transmission line, which causes a periodic modulation of the substrate and device impedance as measured at the reference plane of the connector/probe interface:

$$\underline{Z_{\mathrm{in}}} = Z_0 \frac{\underline{Z_{\mathrm{L}}} + iZ_0 \tan(\beta l)}{Z_0 + i\underline{Z_{\mathrm{L}}} \tan(\beta l)} \qquad (2)$$

$\beta = \frac{2\pi}{\lambda}$, where a propagation speed of $v_{\mathrm{p}} = 0.77c_0$ is taken based on the specifications of the RF cables used, with the speed of light in vacuum corresponding to c_0. The impedance of the device with substrate coupling in parallel is:

$$\underline{Z_{\mathrm{L}}} = \frac{R_{\mathrm{dev}}\left(R_{\mathrm{sub}} + \frac{1}{i\omega C_{\mathrm{tot}}}\right)}{R_{\mathrm{dev}} + R_{\mathrm{sub}} + \frac{1}{i\omega C_{\mathrm{tot}}}} \qquad (3)$$

The real part of $\underline{Z_{\mathrm{in}}}$ was fitted to Supplementary equation 2, where C_{tot}, R_{sub} and l are fitting parameters. The fitting is shown in Supplementary Figs. 1d and e as a green line. The dashed black line shows $\underline{Z_{\mathrm{L}}}$, which reveals, that the shunting capacitance is dominant at low frequencies. The substrate resistance limits the shunting at higher frequencies. The values for C_{tot}, R_{sub} and l for $n = 0$ % are thus determined to be:

$$C_{\mathrm{tot}} = 11.66\ \mathrm{pF}$$

$$R_{\mathrm{sub}} = 317.59\ \Omega$$

$$l = 2.12\ \mathrm{cm}$$

which are close to the values we had estimated based on the device geometry. The peak of the reflectance in Supplementary Fig. 1b at around 12 GHz is caused by a circuit resonance that

has not been considered in this circuit model. However, our analysis of the spin pumping and field like torque contributions is for $f = 9\ GHz$, where this resonance peak is not relevant. One can calculate the transmitted power and the total current through the device after determining C_{tot}, R_{sub}, R_{dev} and using the applied power P:

$$P_{\mathrm{trans}} = \left(1 - \underline{\Gamma\Gamma^*}\right)P \qquad (4)$$

$$I_{\mathrm{rf,tot}} = \frac{R_{\mathrm{sub}} + \frac{1}{i\omega C}}{R_{\mathrm{dev}} + R_{\mathrm{sub}} + \frac{1}{i\omega C}} \sqrt{2} \sqrt{\frac{P_{\mathrm{trans}}}{\left|\underline{Z_{\mathrm{L}}}\right|}} \qquad (5)$$

Supplementary Fig. 1f shows the calculated total RF current through the device versus oxygen content.

We now focus on calculating the influence of spin pumping. The spin pumping will reduce the symmetric part of equation 1 (main paper) and is always opposite to V_0S. The value of the spin pumping voltage is given by:

$$V_{\mathrm{sp}} = \theta_{\mathrm{LW}}^{\mathrm{eff}} \frac{ew\lambda_{\mathrm{S}}R_{\mathrm{dev}}}{2\pi} \tanh\left(\frac{d}{2\lambda_{\mathrm{S}}}\right) G_{\mathrm{eff}} \langle \mathbf{m} \times \dot{\mathbf{m}} \rangle \qquad (6)$$

$$\langle \mathbf{m} \times \dot{\mathbf{m}} \rangle = 2\pi f {\phi_{\mathrm{p}}}^2 \sin\varphi \sqrt{\frac{H_0}{H_0 + M_{\mathrm{eff}}}} \qquad (7)$$

$$\phi_{\mathrm{p}} = \frac{1}{dR/d\varphi} \frac{2}{I_{\mathrm{rf,tot}}} V_0 \sqrt{S^2 + A^2} \qquad (8),$$

where w is the device´s width (10 μm), $\langle \mathbf{m} \times \dot{\mathbf{m}} \rangle$ describes the precession, and ϕ_{p} the maximum precession angle, which can be calculated from $dR/d\varphi$ at 45°, the total RF current through the device and the symmetric (S) and asymmetric (A) components. The spin diffusion length λ_{S} was assumed to be 3 nm.[4] $dR/d\varphi$ was determined by measuring the spin Hall magnetoresistance/AMR of the devices. Supplementary Fig. 2a shows the measured $dR/d\varphi$ for $\varphi = 45°$ versus oxygen content. Supplementary Fig. 2b reveals the V_{sp} in comparison to the symmetric part and there is a considerable contribution due to spin pumping for $n = 0\ \%$

and $n = 44$ %, which correspond to the lowest and highest oxygen contents we study here, respectively.

To account for the field like torque one has to correct the β_{Oe} factor of $d/2$ in equation 6 of the main paper. The $d/2$ arises from the Oersted field which is: $H_{\mathrm{rf}} = J_{\mathrm{c,W(O)}} d/2$. We have to add to this field, a field that arises from the field-like STT: $H_{\mathrm{tot}} = H_{\mathrm{rf}} + H_{\mathrm{FL}} = \beta_{\mathrm{Oe+FL}} J_{\mathrm{c,W(O)}}$, from which follows that

$$\beta_{Oe+FL} = \frac{d}{2} + \frac{H_{FL}}{J_{c,W(O)}} \qquad (9)$$

β_{Oe+FL} factor can be deduced from the asymmetric part: $A = H_{tot}\sqrt{1 + M_{eff}/H_0}$. Thus,

$$\beta_{\mathrm{Oe+FL}} = \frac{A}{J_{\mathrm{C,W(O)}}\sqrt{1 + M_{\mathrm{eff}}/H_0}} \qquad (10)$$

The current through the W(O) layer was determined from $I_{\mathrm{rf,tot}}$ by using the resistance ratio of the two layers and the device dimensions. Supplementary Fig. 2c shows $I_{\mathrm{rf,tot}}$ in comparison to the current through the W(O) layer $I_{\mathrm{rf,W(O)}}$. For determining A from the V_{mix} fitting, one has to calculate V_0 (equation 2, main paper), where again knowledge of $dR/d\varphi$ is necessary. $(df/dH_{\mathrm{ext}})|_{H_{\mathrm{ext}}=H_0}$ was determined from the Kittel formula. Supplementary Fig. 2d shows $\beta_{\mathrm{Oe+FL}}$ in comparison to the β_{Oe}. Large field like contributions are observed at $n = 0$ % and $n = 12.1$ % where the deviation between $\theta_{\mathrm{LS}}^{\mathrm{eff}}$ and $\theta_{\mathrm{LW}}^{\mathrm{eff}}$ analysis is largest (Fig. 3c, main paper). At $n = 12.1$ %, one expects a lower $\beta_{\mathrm{Oe+FL}}$ compared to β_{Oe}, which is also reflected by Fig. 2d. Thus, one can correct the line shape analysis $\theta_{\mathrm{LS,corr}}^{\mathrm{eff}}$ by taking into account the spin pumping voltage and field like torque contributions as follows:

$$\theta_{\mathrm{LS,corr}}^{\mathrm{eff}} = \frac{S - \mathrm{sgn}(\theta_{\mathrm{LW}}^{\mathrm{eff}})|V_{\mathrm{sp}}|}{A} \frac{2e\mu_0 M_{\mathrm{S}} t}{\hbar} \beta_{\mathrm{Oe+FL}} \sqrt{1 + \frac{M_{\mathrm{eff}}}{H_0}} \qquad (11)$$

Supplementary Fig. 2e shows the $\theta_{\mathrm{LS,corr}}^{\mathrm{eff}}$ (green) in comparison to $\theta_{\mathrm{LS}}^{\mathrm{eff}}$ and $\theta_{\mathrm{LW}}^{\mathrm{eff}}$. The corrections do work well at low n, but do so show deviations at larger n. Supplementary Fig.

2f shows to which values the total RF current has to be adjusted in order to equate $\theta_{\mathrm{LW}}^{\mathrm{eff}}$ and $\theta_{\mathrm{LS,corr}}^{\mathrm{eff}}$. The green dashed line in Fig. 2e shows $\theta_{\mathrm{LS,corr}}^{\mathrm{eff}}$ if one uses the adjusted current values from Fig. 2f. The average deviation between the calculated total RF current to the adjusted current is 9.38 % and the deviation is largest for the first and last samples, where spin-pumping is more significant. Generally speaking, the deviation between the measured and adjusted currents in Fig. 2f is not so significant, implying that $\theta_{\mathrm{LS,corr}}^{\mathrm{eff}}$ is very sensitive to small current variations. Thus, the difference between $\theta_{\mathrm{LS}}^{\mathrm{eff}}$ and $\theta_{\mathrm{LW}}^{\mathrm{eff}}$ can be accounted for based on these corrections.

Supplementary Note 2: Effective spin mixing conductance

Supplementary Fig. 3 shows the effective spin mixing conductance versus oxygen content and a relatively constant behaviour is observed. The data is obtained by measuring change in the Gilbert damping with the presence of the W(O) layer using conventional strip-line ferromagnetic resonance (FMR):

$$G_{\mathrm{eff}} = \frac{4\pi M_{\mathrm{S}} t}{g\mu_{\mathrm{B}}}\left(\alpha_{\mathrm{W(O)|CoFeB}} - \alpha_{\mathrm{CoFeB}}\right) \qquad (12),$$

where g is the Lande g-factor, μ_{B} is the Bohr magneton, $\alpha_{\mathrm{W(O)|CoFeB}}$ is the Gilbert damping of the W(O) | CoFeB film, and α_{CoFeB} is for the film with CoFeB only.

Supplementary Note 3: XRD of ST-FMR films (60 Å) and thick (500 Å) calibration films

Supplementary Fig. 4a and b reveals the XRD of the ST-FMR films, which are used for the SHA measurement. Since the CoFeB is amorphous there is only an influence of the W(O). The signal-to-noise ratio is much smaller compared to the XRD of multilayers (Fig. 4a; main paper). Supplementary Figs. 4c and d, show the XRD for 50 nm thick (Si substrate | SiO_x (25) | W(O) (50)) W(O) films. For pure tungsten, only the α-phase is present, whereas by comparison, for the 6 nm counterpart films of the same kind, some amount of the β-phase was

also present in the film (Fig. 4a, main paper). Moreover, we see that for a small amount of oxygen gas, the β-phase is formed and at $Q = 1.4$ %, there is sudden jump towards the formation of nano-crystalline W(O).

Supplementary Note 4: Resistance versus Temperature for W(O) for thick (50 nm) and thin (6 nm) films

We measured the resistance of W(O) (Si substrate | SiO_x (25) | W(O) (50) for thick films and Si substrate | SiO_x (25) | W(O) (6) | TaN (2) for thin films) films as a function of temperature from 2K to room temperature. The resistance is normalized with respect to the resistance as measured 300 K. For 100 nm thick β-W films, the superconducting transition temperature has been reported to be between 2 K and 3 K.[5] For the thin films (6 nm) studied here (Supplementary Figs. 5a and 5b), whilst we do not directly observe the superconducting transition temperature, we do observe a sudden decrease in the resistivity indicative of a superconducting transition temperature that is below 2 K. The highest resistivity decrease for these thin films is reached for $n = 25.5$ % ($Q = 1.2$ %). Moreover, we note that the resistivity goes up with decreasing temperature before it turns superconducting. This increase in resistivity is more significant for higher oxygen gas flow and for $n = 44$ % ($Q = 3$ %), the resistivity actually increases abruptly at low temperatures, and so becomes highly resistive instead of superconducting. The resistance increase with temperature in general could be explained by the amorphous nature of the material and hopping transport mechanisms. Thermally activated electron transport may also account for the increase in resistance with decreasing temperature.[6]

We also performed similar experiments for 50 nm thick films (Supplementary Figs. 5c and 5d). For these samples, a clear superconducting transition is seen at $T \approx 2$ K for several oxygen concentrations.

Supplementary Note 5: Inhomogeneous broadening

Supplementary Fig. 6 shows the inhomogeneous broadening for various oxygen concentrations. We note that it is rather small and uncorrelated to changes that we observe in the SHA, and hence does not have an impact on the quantitative determination of the effective SHA.

Supplementary References

1. Nan, T. *et al.* Comparison of spin-orbit torques and spin pumping across NiFe/Pt and NiFe/Cu/Pt interfaces. *Phys. Rev. B* **91,** 214416 (2015).

2. Mellnik, a. R. *et al.* Spin-transfer torque generated by a topological insulator. *Nature* **511,** 449–451 (2014).

3. Wang, Y., Deorani, P., Qiu, X., Kwon, J. H. & Yang, H. Determination of intrinsic spin Hall angle in Pt. *Appl. Phys. Lett.* **105,** 152412 (2014).

4. Hao, Q. & Xiao, G. Giant Spin Hall Effect and Switching Induced by Spin-Transfer Torque in a W/$Co_{40}Fe_{40}B_{20}$/MgO Structure with Perpendicular Magnetic Anisotropy. *Phys. Rev. Appl.* **3,** 34009 (2015).

5. Bond, W. L. *et al.* Superconductivity in films of tungsten and other transition metals. *Phys. Rev. Lett.* **15,** 260–261 (1965).

6. Hao, Q., Chen, W. & Xiao, G. Beta (β) tungsten thin films: Structure, electron transport, and giant spin Hall effect. *Appl. Phys. Lett.* **106,** 182403 (2015).
